# Rutting Behavior of Asphalt Surface Layers Designed for Solar Harvesting Systems

**DOI:** 10.3390/ma16010277

**Published:** 2022-12-28

**Authors:** Marco Pasetto, Andrea Baliello, Giovanni Giacomello, Emiliano Pasquini

**Affiliations:** Department of Civil, Environmental and Architectural Engineering, University of Padova, Via Marzolo 9, 35131 Padova, Italy

**Keywords:** road pavement, asphalt mix, rutting, permanent deformation, high in-service temperature, rut depth, asphalt solar collector, steel slag

## Abstract

Solar harvesting systems applied to asphalt roads consist of pipes or coils installed a few centimeters below the asphalt pavement surface. They work thanks to a circulating fluid able to collect the heat coming from solar irradiation of the pavement surface and convert it into thermal gradients that can be used for electric energy supply. Specific attention must be paid to the design of the asphalt mixtures comprising the system. In this sense, the high in-service temperature rutting potential is one of the main issues to be assessed in such applications since the thermal optimization of asphalt mixes could lead to excessively deformable materials. The present study is a part of a wider research area aimed at developing an efficient asphalt solar collector. Here, a laboratory mixture-scale investigation is proposed to verify the anti-rutting potential of specific asphalt layers that were initially designed based on thermal properties only. Repeated load axial and wheel tracking tests are carried out on limestone- and steel slag-based bituminous mixtures. Overall, the tested layers were not fully able to satisfy the permanent deformation acceptance criteria; in this regard, possible improvements in terms of mix constituents and properties are ultimately addressed.

## 1. Introduction

Permanent deformation on road asphalt pavements is a distress suffered by the surface layers at high in-service temperatures and low-speed traffic [1]. It is widely studied because of the issues in ride quality (evenness) and safety (e.g., aquaplaning) of pavements affected by such non-negligible depressions along the wheel paths (i.e., ruts) [2]. 

Permanent deformation arises mainly because of both the visco-elastic thermal-dependent response of the bituminous binder, which softens at high temperatures, and the aggregate skeleton properties (i.e., maximum aggregate size and granulometric distribution), which hinder possible internal rearrangements due to traffic load applications [3,4]. Actually, rutting phenomena also depend on environmental conditions and traffic characteristics (load magnitude, volume, speed, etc.) [5]. Therefore, a multi-scale analysis approach aimed at investigating the rheological and mechanical behaviors of asphalt mixes is strongly suggested to strictly characterize the overall pavement anti-rutting potential. This is typically investigated by studying the materials at different scales (namely evaluating the responses of binders, mastics, mortars, and mixtures) and assessing the pavement performance under several environmental conditions (representative of the real in-service characteristics) [3,6].

In this regard, several literature studies provide suitable guidelines to optimize asphalt mixtures against rutting phenomena: they mainly concern the optimization of the lithic skeleton and the volumetric properties of mix (to promote higher internal friction and enhance the aggregate interlock) [7], the use of polymer modified asphalt binders [8], the production of warm [9] or cold [10] mix asphalts, or pavement reinforcement through geogrids [11].

Regardless of the specific testing approach used to analyze permanent deformation resistance, the exhibited rutting accumulation can be summarized by a three-stage structure [12]: a first stage characterized by a decreasing creep rate (i.e., slope of the curve deformation vs. number of applied cycles); a second stage in which permanent strains are accumulated at an approximately constant rate (quasi-constant creep rate) where a combination of densification and shear deformations occurs; and a third stage with an increasing creep rate, in which aggregates are forced to move upheavals to the side, and a shear failure of asphalt mixture occurs.

At the laboratory scale, wheel-tracking tests on lab-produced slabs or repeated axial creep tests on cylindrical specimens [6] are commonly used for material characterization since they are able to reproduce the repeated application of traffic load on pavement surfaces [13]; on the other hand, full-scale accelerated pavement testing systems made by heavy vehicle simulators can be used at field scale [14].

As examples, a Hamburg wheel tracking test was used to study the rutting resistance of hot-mix asphalt with different gradations and asphalt contents and indicated that the properties of the fine aggregate fraction (4.75 mm maximum aggregate size) had a positive influence on the rutting resistance [15]. A wheel tracking laboratory device was also used to discriminate the short-term and secondary rutting effects on various asphalt mixes, demonstrating that mixtures could be characterized mainly based on primary rutting [16]. Asphalt content was correlated with rutting properties, showing that an optimum bitumen dosage could lead to a positive increase in rutting resistance because of an enhanced cohesion between aggregates [17], depending on the thickness of asphalt film coating the aggregates [15]. At mixture scale, a stochastic approach to evaluate the rut depth of hot and warm bituminous mixtures was set up based on wheel tracking tests; it was able to take into account the effect of aging on bitumen visco-elastic properties with respect to rutting phenomena [18]. On the other hand, some literature results highlighted the significance of the angularity of the fine aggregates against rutting [19]. Other researchers observed that the total amount of coarse aggregates had a certain influence on mixture resistance to permanent deformation executing laboratory wheel tracking and cyclic compression tests [20].

Furthermore, the possible use of secondary, recycled, or manufactured aggregates can be considered to enhance rutting resistance while also promoting the asphalt mixture’s sustainability. In fact, the use of a secondary product allows for converting wastes into valuable resources for construction fields [21], avoiding landfill disposals (e.g., approximately 21 million tons of steel slag were produced during the last decade in the United States [22]). In this regard, electric arc furnace (EAF) steel slag, i.e., a waste product coming from the metallurgical processing generated during iron making, can be reused as high-quality aggregate in asphalt mixes thanks to its physical and mechanical properties (hardness, toughness, adhesiveness, and roughness) [23,24,25]. An improved resistance to high-temperature permanent deformations can be achieved thanks to such enhanced properties, which improve aggregate interlocking [26].

The anti-rutting performance must be further investigated for special applications, such as asphalt solar collectors (ASC), i.e., efficient energy harvesting systems able to collect thermal energy due to pavement sunlight irradiations and to convert it in other spendable/renewable energy [27,28]. In this application, energy collection is achieved through the circulation of thermally-optimized fluids through ad hoc coils or pipes installed a few centimeters under the pavement surface (embedded in the wearing course) [29]. As conceived, the temperature concerns could be in these cases more relevant since the operation of such systems is promoted by high-conductive asphalt mixtures able to dissipate the heat collected on the surface through the pavement depth [30]. In this sense, a thermal optimization of ASC asphalt mixes could lead to excessively deformable materials, which could then suffer unacceptable permanent deformation.

Given this background, the present research deals with a laboratory mixture-scale characterization focused on the analysis of the rutting performance of special bituminous mixtures manufactured with steel slag aggregates specifically designed for the construction of an asphalt solar pavement collector.

## 2. Research Objectives and Approach

The manuscript describes an experimental laboratory study investigating the rutting resistance of asphalt mixtures produced with conventional aggregates (limestone) or manufactured aggregates (steel slag) whose characteristics can lead to well-known environmental benefits related to saving and recycling. Additionally, the study of rutting behavior of asphalt concretes is extended to special pavement structures, as the study is part of a wider research program regarding the set-up of a solar collectors in road pavement. In this sense, the paper contributes to the knowledge of the permanent deformation mechanisms in the case of special asphalt systems designed to optimize thermal characteristics (solar heat harvesting), including foreign steel collectors.

Thus, the mechanical feasibility of such bituminous mixtures is investigated to verify their suitability for comprising the asphalt solar collector layers. The analyzed ASC structure was designed in a previous step of the research [31] and was composed of:

- a bottom asphalt layer prepared with an 8 mm maximum aggregate size dense-graded bituminous mixture representative of the existing road pavement;
- a steel collector for a fluid circulation placed at the interface;
- a top 4 cm thick asphalt layer prepared with an 8 mm maximum aggregate size dense-graded bituminous mixture compacted at 4% air voids to maximize the heat transfer within the pavement.

With respect to the previous literature studies, which mainly focused on the thermal performance of the investigated energy harvesting systems, the present research also addresses the rutting resistance of such special asphalt mixtures that were originally designed based on thermal considerations [31] only (by simply matching Italian technical requirements about gradations and void content). In this sense, the asphalt mixes prepared to maximize heat transmission through the asphalt depth were characterized by:

- quite thin thickness (4 cm), to reduce the installation depth of the collector;
- small nominal maximum aggregate size (NMAS) suitable for thin-layers;
- quite low void contents (fixed at 4%), to reduce the insulating air pore structure.

Therefore, the present study aims to verify the permanent deformation resistance and its acceptability (with respect to Italian technical specifications) for asphalt systems (double-layered systems containing a steel coil) reproducing the previously analyzed ASCs. Such experimental research supplements another preliminary research study [32] that aimed to test at a smaller scale the constituent materials of the ASC mixtures (binder-, mastic-, and fine-mortar-scale rheological tests).

**Figure 1:**
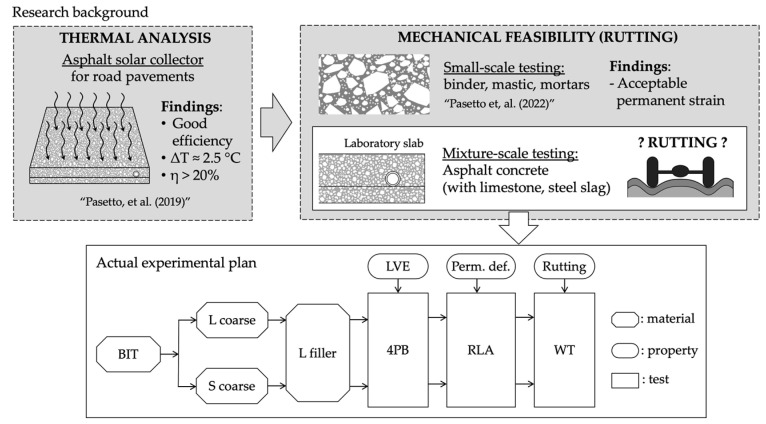
Global research framework and experimental plan [31,32].

The global research framework and the actual experiment plan is summarized in Figure 1 (all the reported acronyms are detailed in the following paragraphs).

## 3. Materials and Methods

### 3.1. Materials

**Figure 2:**
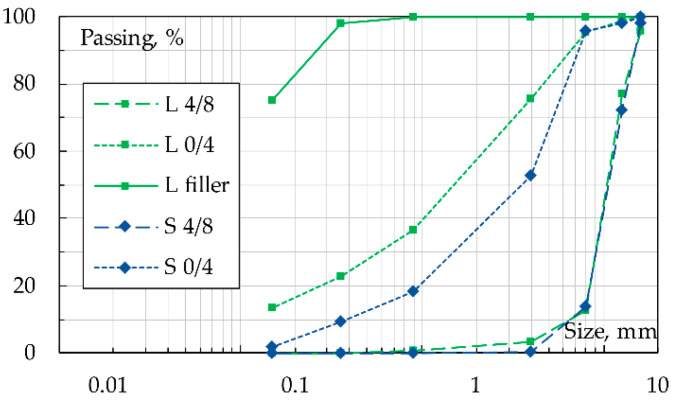
Available stockpiles for the selected aggregates: limestone (*L*) and steel slag (*S*).

As noted, constituent materials for asphalt mixtures where those of the original study [31]. A 50/70 penetration grade plain bitumen (coded as *B*) was utilized as a binder; its physical properties are summarized in Table 1. Two different aggregates available in various stockpiles (Figure 2), i.e., natural limestone (coded *L*) and manufactured/recycled EAF steel slag (coded *S*), were used to produce the asphalt mixtures. S aggregates have been considered in this study since they could be able to enhance the permanent deformation resistance while also providing some improved thermal properties to the investigated ASC. Both *L* and *S* meet the acceptance criteria for paving aggregate (details can be found elsewhere [32]). Steel slag (S) filler was excluded for both technical and economic concerns [33].

### 3.2. Sample Preparation

**Figure 3:**
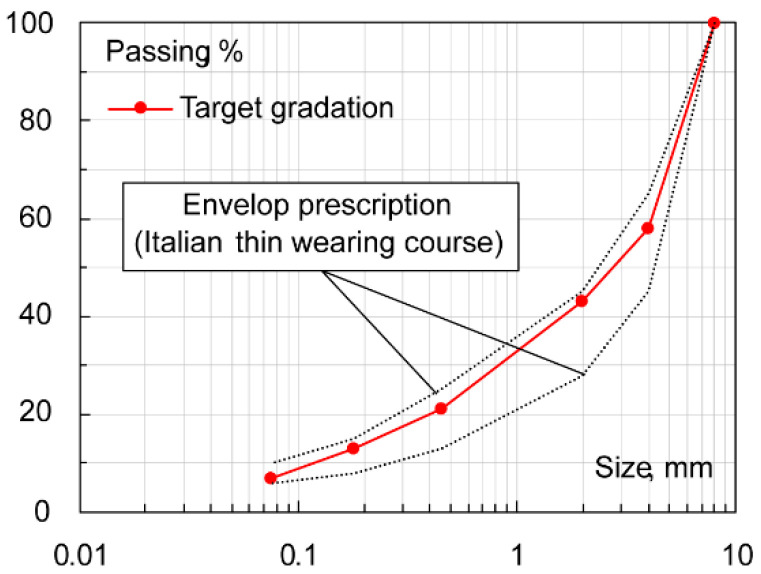
Envelop specifications and target gradation of *ACL* and *ACS* mixes.

Bitumen and aggregates (*L* filler excluded) were oven-heated at 160 °C and blended with an automatic mixer (30 min at 160 °C). The limestone-based asphalt mixture is coded as *ACL*, whereas the steel slag-based bituminous mixture is coded as *ACS*: mixtures’ gradations and mix-design details are shown in Table 2 and Figure 3. Compaction with a Shear Gyratory Compactor–SGC according to EN 12697-31 (device Controls ICT 100-150RB), or a Roller Compactor–RC according to EN 12697-33) (device Controls Dyna-Comp 77-B3602) was carried out to create double-layer cylindrical 150-mm diameter specimens or prismatic slabs (plain dimensions of 300 × 400 mm), respectively.

Samples were obtained through the following steps: (i) compaction of the first layer of asphalt concrete (*ACL* or *ACS*) to simulate an existing new binder course or milled surface; (ii) application of a bituminous emulsion C55B2 as tack coat, dosed at 0.3 kg/m^2^ residual bitumen; (iii) installation of a steel coil/pipe on the emulsified surface; (iv) compaction of the upper layer above the coil/pipe reproducing the top wearing course.

**Figure 4:**
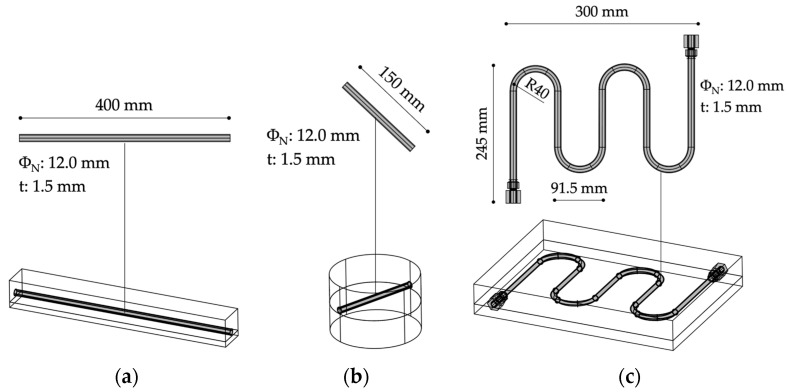
Details about the produced samples: example of PR (**a**), CY (**b**), and SL (**c**).

**Figure 5:**
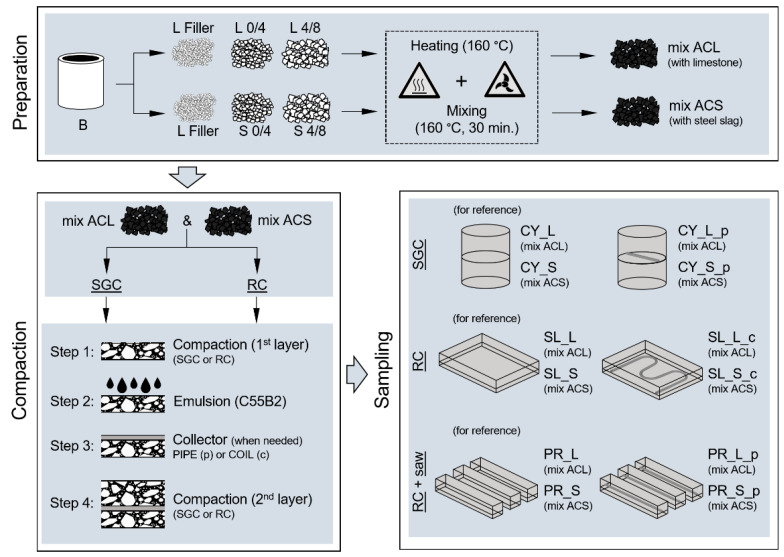
Flowchart of mixtures’ production, compaction, and sampling.

The coils/pipes were made of steel (nominal diameter *Φ_N_* of 12 mm, thickness *t* of 1.5 mm) and constituted the core of the energy harvesting system that, through water circulation, should be able to collect the heat collected by the asphalt pavement. In this regard, among the possible alternatives, plastic pipes were assumed to be incompatible with the high temperatures involved during compaction of the bituminous mixture, whereas copper systems were supposed to be not economically sustainable. Combining *ACL* and *ACS* mixes, different specimen shapes, thicknesses, and pipe configurations, various laboratory samples were created (also by sawing the slabs). For all sample sets, double-layered reference systems without steel collectors were produced for comparison purposes. All the details are reported in Table 3, whereas Figure 4 schematizes the shape of such samples. Finally, Figure 5 schematizes the mixtures’ production, compaction, and sampling.

### 3.3. Testing Methods

The anti-rutting performance at mixture-scale was preliminarily assessed by determining the main visco-elastic properties of the produced mixes. The prismatic samples (*PR-L-p* and *PR-S-p* with straight pipe, *PR-L* and *PR-S* without steel pipe) were tested in a four-point-bending (4PB) configuration applying, in strain-controlled mode, a sinusoidal displacement (50 μstrain amplitude) with test frequencies varying from 0.1 to 20 Hz. After four hours of conditioning time, five test replicates were executed for each sample for the sake of data reliability. Results were expressed in terms of the norm of complex modulus |E*| and phase angle *Φ* measured at the 100th cycle according to EN 12697-26/Annex B. Prismatic samples were tested at three temperatures (5, 15, and 25 °C). According to the Williams–Landel–Ferry approach [34], such data were used to construct the master curves at the reference temperature 15 °C.

Rutting potential was then directly evaluated by carrying out repeated load axial (RLA) tests according to the BS DD 226 standard. This test was carried out on the produced cylindrical double-layered samples (*CY-L-p* and *CY-S-p* with straight pipe, *CY-L* and *CY-S* without steel pipe). Three replicates for each sample were tested. The axial load was applied in unconfined mode using a 150-mm diameter upper plate on specimens with the same diameter. After a pre-conditioning time of four hours, samples were subjected, in stress-controlled mode, to a cyclic square stop-pulse pressure with a frequency of 0.5 Hz (i.e., 1.0 s loading time and 1.0 s rest period) for a total duration of 10,000 pulses. Three levels of stress (300, 350, 400 kPa) were replicated at a temperature of 60 °C. This testing temperature was selected according to common technical specifications since it is representative of the maximum temperature achievable by standard pavements during the hottest periods. Even though the studied ASC is able to achieve lower service temperatures due to the cold fluid circulating within the system, 60 °C was selected as the testing temperature to precautionarily take into account possible inactivity periods of the harvesting system. Material behaviors were analyzed to monitor the evolution of the cumulative axial strain *ε_a_* as a function of the number of loading cycles *N*. This relationship was expected to be described by the already-mentioned three-party curve in which: (i) the first part exhibits a decreasing creep rate (i.e., slope of the curve); (ii) the second phase shows a quasi-constant creep rate; and (iii) the third part presents a further increase of the creep rate up to the physical failure. Rutting potential of the tested mixtures was estimated in terms of creep rate *f_c_* of the quasi-linear second part of the curve. Flow number *FN* (i.e., the cycle at which tertiary flow starts) was also calculated as a key indicator of the rutting resistance of asphalt mixtures [12].

Finally, wheel tracking (WT) tests were performed according to the EN 12697-22 standard (three replicates for each sample). In this case, 300 × 400 mm base SL slabs were studied by applying repeated passes of a moving pneumatic tire (load applied on specimen equal to 700 N) at a temperature of 60 °C (4 h of pre-conditioning time was selected to ensure homogeneous thermal conditions of specimens). According to the reference standard, a loading rate of 26.5 cycles/min (53 passes/min) was selected up to 10,000 cycles; the rut depth occurring in the central part of the specimens’ surfaces was continuously measured through a linear variable differential transformer (LVDT). The obtained data were then analyzed by considering the average of three test repetitions for each of the tested double-layered systems (*SL_L* and *SL_L_c* prepared with *ACL* mixtures, *SL_S* and *SL_S_c* obtained with *ACS* mixes).

**Figure 6:**
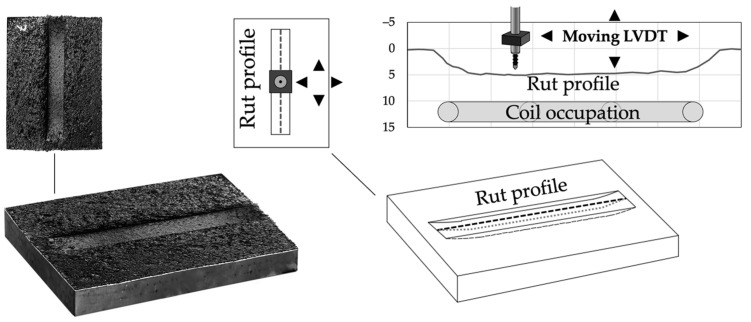
Rut profile measuring equipment.

Furthermore, the produced systems were also analyzed through laboratory equipment constituted by a mountable frame free to move in three dimensions and equipped with an LVDT measuring device. It allowed for reconstructing the longitudinal profile of the ruts produced (Figure 6). Such measurements were specifically addressed by considering (in comparative terms) the possible effects due to the steel coils under the surface. The evolution of loading cycles of rut profiles was also assessed for each sample (three replicates per type) using the measurement device at different test stages (after 2500, 5000, 7500, and 10,000 cycles).

## 4. Experimental Results and Analysis

### 4.1. Laboratory Results

**Figure 7:**
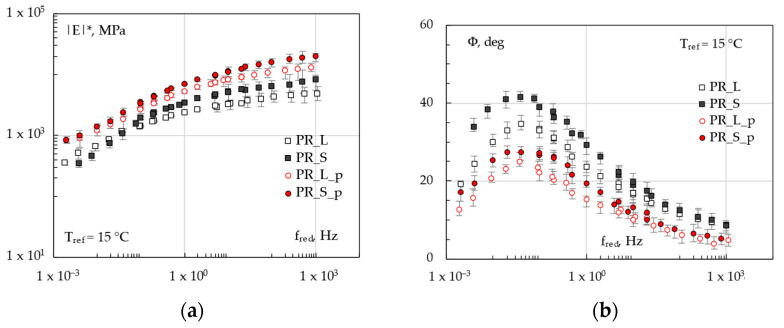
Master curves of mixtures at 15 °C (4PB test): |E*| (**a**) and *Φ* (**b**).

The visco-elastic properties of the mixtures are summarized in Figure 7, which shows the master curve of |E*| and *Φ* at the reference temperature of 15 °C calculated based on average 4PB test results for each sample (limestone or steel slag-based specimens, with or without pipe). First, a clear stiffening effect due to the use of steel slag aggregates was observed with respect to the corresponding limestone-based systems (*PR_S* and *PR_S_p* in comparison with *PR_L* and *PR_L_p*, respectively). This impact of the steel slag aggregates, already observed by several other researchers who conducted similar studies [33,35,36,37], is likely due to the stronger particle interaction in the case of rougher and harder S aggregates. 

As expected, the presence of the pipe conferred higher rigidity to the systems and seemed to produce a more noticeable increase in |E*|, regardless of the mixture type. Within the analyzed temperature range, the frequency dependence is similar for each material, resulting in an upward-like shift of the stiffness master curves when considering the manufactured aggregate, the metallic element within the samples, or the combination thereof. 

As far as the phase angle *Φ* is concerned, all curves are characterized by the bell-shaped trend commonly observed for asphalt mixes at the obtained frequency range and hypothesized using the most known predicting models for asphalts [38]; it is also worth noting that the location peak (maximum *Φ* value) seemed to be very similar for all the mixtures (at about 0.5 Hz), nevertheless exhibiting a predominant elastic response (*Φ* always < 45 °C). Moreover, from the physical point of view, the effects of *_S* (slag) and *_p* (pipe) on viscosity is opposite. Steel slag *Φ* curve (*PR_S*) is higher than the corresponding one (*PR-L*), suggesting that the manufactured aggregate led to a general stiffening along with more viscous responses of the mixes. According to the previous research step [32], this could suggest an altered bitumen-aggregate interaction and could likely be attributed to a possible lower bitumen-slag affinity, also reported by other literature studies on similar materials [33]. In this perspective, it could be supposed that a lower bitumen-aggregate affinity could slightly weaken the adhesion properties of a mixture, resulting in different visco-elastic responses detected by the experimental tests [39]. On the other hand, the pipe installation resulted in an increase in the elasticity of the systems (lower phase angles), overbalancing the effects due to the steel slag (see *PR_S_p* curve). Nevertheless, all curves were characterized by a rather similar shape and seemed to be obtained through vertical-like shifts, indicating a very similar frequency dependence of *Φ* and thus an analogous visco-elastic behavior at the different testing temperatures. Overall, given the complexity of the visco-elastic responses at 15 °C and the mutual consequences of *_S* and *_p* on |E*| and *Φ*, it was not possible to establish unequivocally if the general increase of stiffening itself was beneficial in mitigating the rutting potential at high in-service temperature.

**Figure 8:**
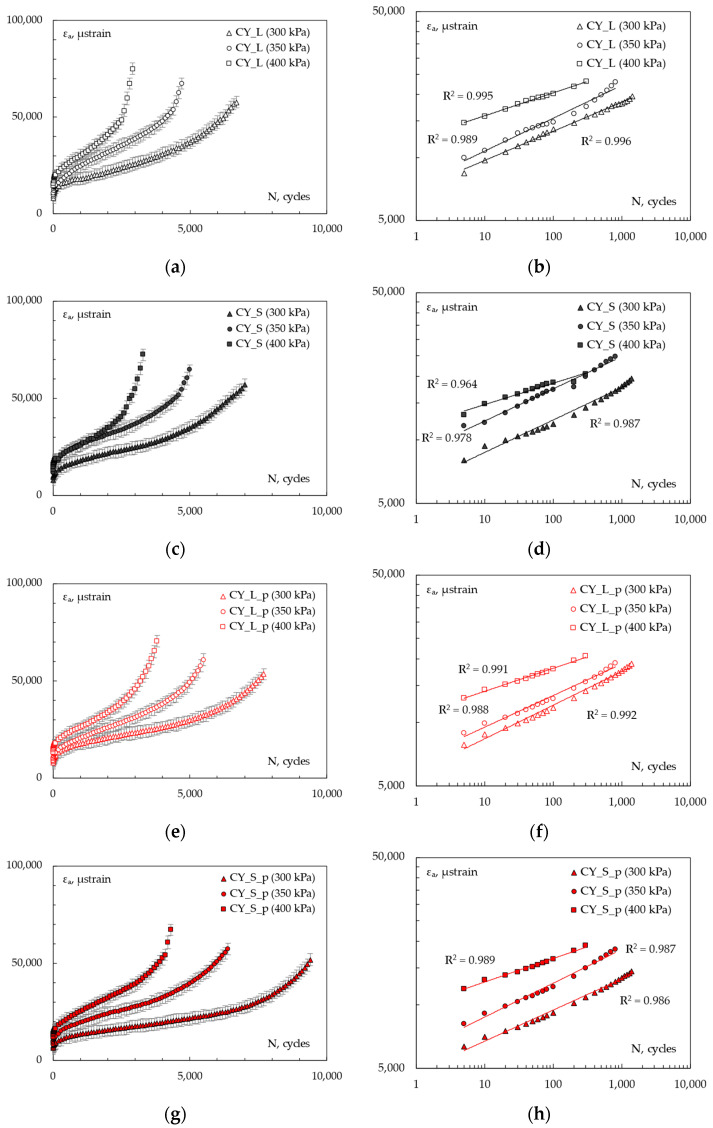
Cumulative axial strain evolution. Standard representation and log–log plot for *CY_L* (**a**,**b**), *CY-S* (**c**,**d**), *CY_L_p* (**e**,**f**), *CY_S_p* (**g**,**h**).

RLA test results at 60 °C are hereafter discussed. The cumulative axial strain vs. the number of loading cycles obtained for each mixture are illustrated in Figure 8. In particular, the diagrams on the left (Figure 8a,c,e,g) were drawn in linear scale clearly representing the abovementioned three-stage behavior, whereas the plots on the right (Figure 8b,d,f,h) report the same data using a log–log scale, allowing for a more effective representation of the second-stage part with power–law regressions expressed by the well-known Equation (1).

*ε_a_* = *k*_1_
*N^k^*^2^ (1)

where *k*_1_ and *k*_2_ are fitting coefficients and denote the permanent strain at the beginning of the text and the rate of change of the permanent strain as a function of loading cycles, respectively.

**Figure 9:**
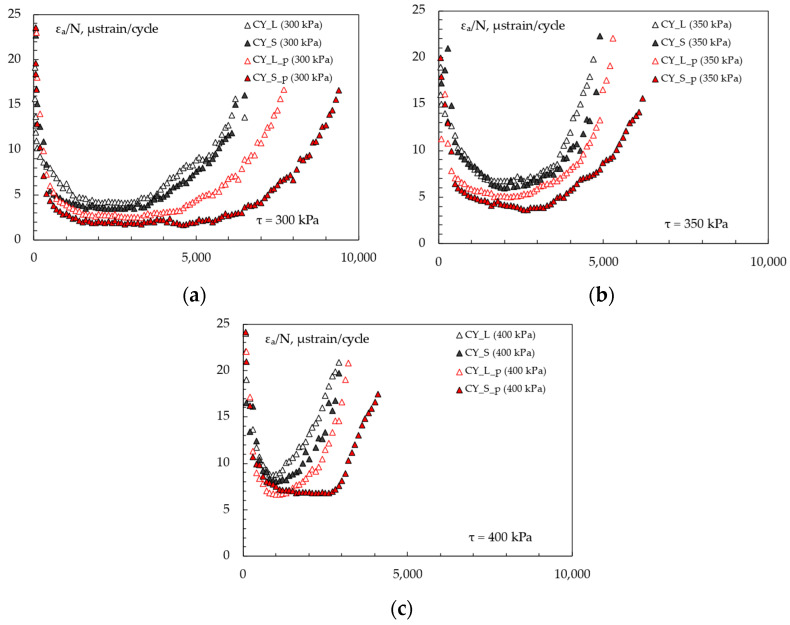
Rate of change in *ε_a_* vs. *N* for *FN* calculation: 300 (**a**), 350 (**b**), and 400 (**c**) kPa.

The following Figure 9 shows the rate of change in permanent strain vs. loading cycle, expressed at different stress levels; it is used to identify the flow number *FN* as the minimum of the plotted curves. All the numerical results (second-stage creep rates *f_c_*, *k*_1_ and *k*_2_ coefficients, and flow numbers *FN*) are summarized in Table 4. RLA tests clearly show the main stiffness-related findings, since the permanent strain accumulations were inversely proportional to the |E*| of mixes. Thus, at a given stress level, steel slag-based samples were less strain-susceptible than the corresponding limestone-based ones. The pipe-containing specimens demonstrated lower overall deformations with respect to the plain ones. 

Such behaviors were also highlighted by the calculated parameters described above. Regardless the applied stress, creep slope *f_c_* decreased when manufactured aggregates were used in the lithic skeleton or when the metallic element was introduced within the samples. Since this is considered a symptom of a lower susceptibility to shear deformations and lateral flows (longer second-stage creep phases), the samples, including both steel slag and the coil (*CY_S_p*), could be considered the most rutting-resistant among the ones analyzed. The selected experimental stress levels (300, 350, 400 kPa) led to clearly distinct three-phase curves because of the unconfined conditions. Based on Figure 8b,d,f,h, acceptable *R*^2^ were obtained, interpolating the *k*_1_ and *k*_2_ regression coefficients in the log–log plots of the second-stage of cumulative axial strain curve; *k*_1_ parameters, representing the log–log intercept of the interpolating lines, decreased when stiffness increased and increased when stress level increased (some exceptions could be attributed to data variability over different test replicates). This was in accordance with the literature that states that, the lower the intercept values: (i) the lower the compliance value; (ii) the higher the mixture stiffness modulus; and (iii) the smaller the permanent deformation. On the other hand, *k*_2_ coefficients are the regression line slope: for a given *k*_1_-value, an increase in the slope *k*_2_ generally means higher permanent deformation. Therefore, stiffer samples should exhibit progressively lower *k*_2_ values, while higher rates of change of the permanent strain should be expected when increasing the applied stress. Actually, such behavior was not remarkable from *k*_2_ numerical values because of possible scattering in the interpolating.

A greater anti-rutting potential when using slags was demonstrated in accordance with previous studies at mortar-scale [32] where the stiffer aggregate led to a delayed strain recovery. *FN* analysis shows more representative aspects since it commonly assumes an excellent correlation with the rut depth development under real service. Since *FN* generally increases as the rut depth decreases, the steel slag mixture can be expected to have higher resistance to the reference ones, confirming the experimental findings at a smaller scale.

Similar considerations can be drawn evaluating the pipe effect; *CY_L_p* and *CY_S_p* samples were characterized by lower *FN* with respect to *CY_L* and *CY_S* ones, regardless the investigated stress. Based on the *FN* numerical results, the increase in stiffness given by the pipe seemed to cause even higher reduction of the rutting potential. As expected, the increase in the stress applied during unconfined RLA testing reduced the *FN* values and anticipated the tertiary flow effects in the mixtures. In general, it should also be remembered that *FN* is affected by the volumetric properties of mixture (e.g., finer gradations can produce mixtures with higher *FN*, even though stress is carried by coarse aggregates [40]); in this regard, 4% of target voids could promote the *FN* increase.

**Figure 10:**
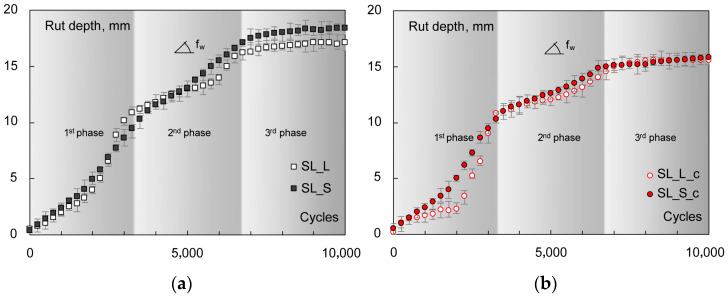
Wheel track results on central slab points: rut depth vs. loading cycles for *SL_L*, *SL_S* (**a**) and *SL_L_c*, *SL_S_c* (**b**).

Figure 10 illustrates the wheel tracking test results in terms of evolution of rut depths for the different mixtures. Materials are grouped in two graphs for the sake of clarity: slabs without (Figure 10a) or with (Figure 10b) the presence of the steel coil. The vertical error bars represent the result’s variability among the different test replicates.

Since rutting data seemed to assume a sort of three-phase trend, some considerations about the rutting resistance of the structures were based on the analysis of the rutting rates *f_w_* of the different phases (*f_w_* was calculated through linear interpolations characterized by high coefficient of determination *R*^2^ for each phase). Table 5 summarizes the main numerical findings related to this elaboration (rutting rate *f_w_* and coefficient of determination *R*^2^), and the final rut depth was attained after 10,000 cycles.

Based on Figure 10, the effect of steel slag is not well defined (depending on the phase, rutting is slightly greater or lower with respect to that of the corresponding reference mix); however, considering the data scattering due to test replicates (see error bars), comparable rutting potential between the different systems can be admitted.

Instead, the stiffening effect given by the coil led to a lower rut depth accumulation for every mix type. Considering the slope coefficients (Table 5), all mixtures presented *f_w_* values with the same order of magnitude at every phase; thus, the effect of steel slag and coil was believed to affect mainly the final rut depth rather than its evolution over time.

**Figure 11:**
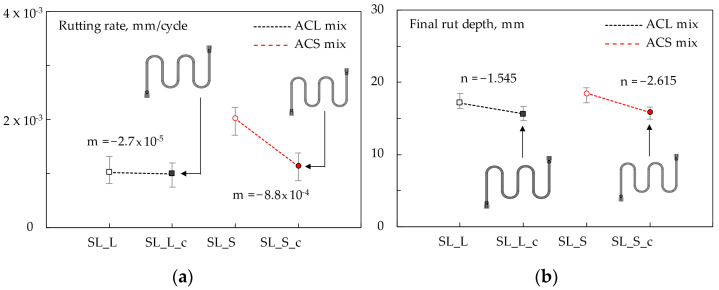
Second-phase rutting rates (**a**) and final rut depths (**b**).

Grouping the rutting rates *f_w_*, the influence of the steel coil can be evaluated. By linear interpolation of the data from the same mixture (with or without coil), it was possible to calculate a slope *m* representing the rutting rate sensitivity with respect to the metallic element placed at the system interface. With a similar approach, mixtures can be grouped with respect to the final rut depths exhibited at the end of the wheel tracking test. Again, the slope of the regression line (*n*) can represent the effect of the coil on the final permanent strain accumulation. The following Figure 11 summarizes this approach (as an example, *m* was referred to in the second phase of the rutting curves). As observed (Figure 11a), the *ACL* mix *m*-value was lower than the corresponding *ACS* slag-based mix. However, it is worth noting that coil-containing specimens (*SL_L_c* and *SL_S_c*) attained very similar *f_w_* values, suggesting the sustainable option of effectively using the studied secondary aggregate.

As far as final rut depths (Figure 11b) are concerned, the coil within the *ACS* mix seemed to exhibit a slightly more evident effect, with *SL_L_c* and *SL_S_c* again showing very similar results.

**Figure 12:**
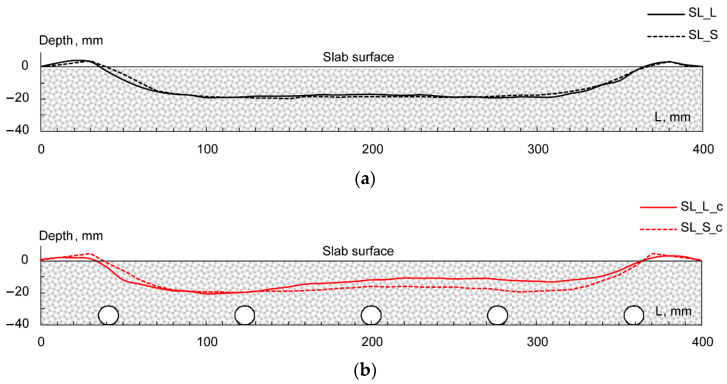
Rut profiles at 10,000 cycles for *SL_L*, *SL_S* (**a**) and *SL_L_c*, *SL_S_c* (**b**).

Further remarks can be made about the evolution of longitudinal rut profiles. Figure 12 depicts the profiles attained at the end of the test (i.e., after 10,000 loading cycles); for the sake of clarity, data have been grouped by mixture type. Analyzing the *ACL* and *ACS* slabs without the coil, the rut profile along the slab centerline demonstrated once again the similar performance of such systems (see Figure 12a). On the other hand, *SL_S_c* specimens showed greater localized depth with respect to *SL_L_c* (right portion of Figure 12b). However, this result can be eventually attributed to the compliance of the measuring apparatus, since the maximum strain accumulated by the two systems is almost superimposed in the left side of the samples. Above all, it is worth noting that no differential rutting was detected (i.e., all profiles were not influenced by the coil presence and configuration), suggesting no negative influence on the rutting behavior of ASC. 

**Figure 13:**
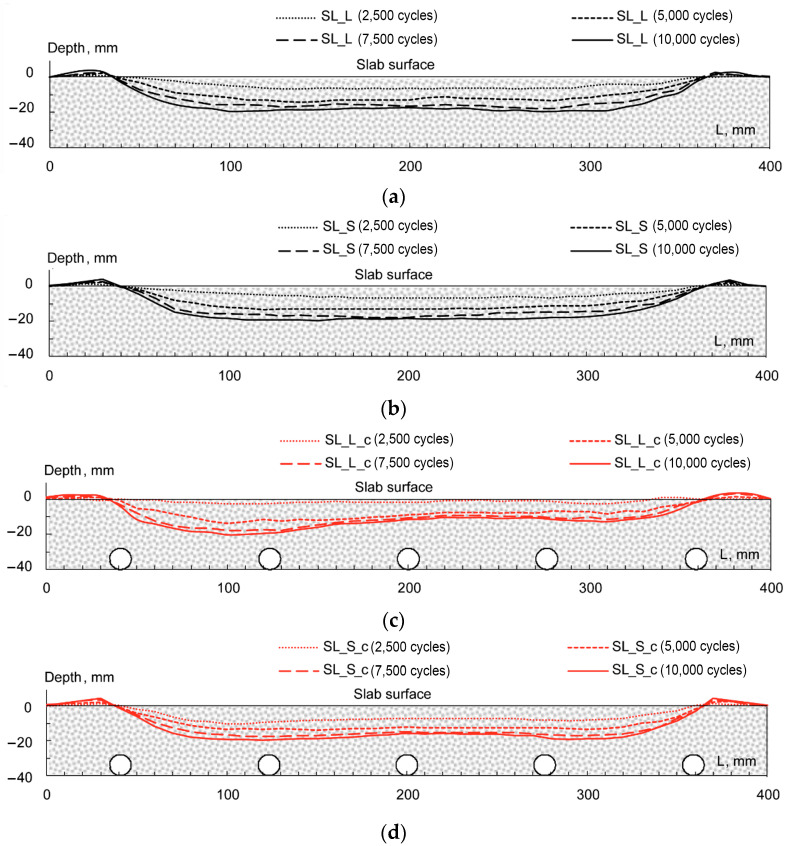
Rut profiles vs. loading cycles: *SL_L* (**a**), *SL_S* (**b**), *SL_L_c* (**c**), SL_S_c (**d**).

Moreover, the analysis of the rutting profiles at different test stages (2500, 5000, 7500 and 10,000 loading cycles) is given in Figure 13. Except for very few outliers, similar strain evolutions can be evinced by comparing the corresponding mixtures at any cycle step (*SL_L* vs. *SLS*, *SL_L_c* vs. *SL_S_c*). This was in accordance with previous findings stating that, regardless of the final amplitudes, rutting depths develop with similar time dependence. Once more, differential (coil-related) rutting was not detected at any cycle step (see 2500, 5000, 7500 cycle profiles).

### 4.2. Discussion

On one hand, the laboratory experimental observations suggest that the substitution of natural aggregates with steel slags did not cause noticeable rutting issues; thus, its use can be promoted to pursue the well-known sustainability benefits without affecting the mechanical suitability of the asphalt mixes. On the other hand, the presence of the metallic coil led to a clear stiffening effect that can be considered beneficial in preventing high-temperature rutting potential.

However, it is worth highlighting that a very high stiffness could be detrimental at intermediate and/or low service temperatures where enhanced ductility and stress-relieving characteristics are required to avoid early cracking phenomena.

At any rate, the presence of a foreign element such as the collector at the interface did not seem to cause any evident permanent deformation issues, providing a structural contribution (i.e., plain stiffness increase). Hence, no substantial adhesion/interface criticisms resulting in detachments or failures could be hypothesized. Furthermore, no differential rutting due to the presence of the coil was observed.

Thus, the obtained experimental findings were promising in view of possible real scale applications of the energy harvesting system that will likely imply greater and widely-spaced coils.

Nevertheless, some doubts concerning the effective permanent deformation resistance of these asphalt mixes still exist since they were specifically designed for the realization of the harvesting systems. In other words, rutting susceptibility seemed to be still uncertain in view of the material properties, mainly designed based on thermal-related aspects arising from previous research [31]. In this regard, consolidated literature demonstrates that a small nominal maximum aggregate size (8 mm in this case) together with the use of a high dosage of a plain bituminous binder generally involve permanent deformation issues [12,41].

Actually, the permanent deformation resistance of the investigated materials was rather poor in absolute terms. As an example, *WTS* parameters (wheel-tracking slope defined in EN 12697-22) calculated for *SL_L*, *SL_S*, *SL_L_c*, and *SL_S_c* were equal to 0.829, 1.072, 0.718, and 0.646 mm/10^3^ cycles, respectively. Such experimental results are basically not acceptable based on common technical specifications for bituminous mixtures that suggest a maximum accepted *WTS* of 0.8 ÷ 1.0. Therefore, 8 mm NMAS allows reduced asphalt thickness covering the coil *c_t_*, thus enhancing the heat collection while weakening the permanent deformation resistance, and thus eventually affecting pavement functionality since *c_t_* could dangerously decrease because of noticeable ruts.

**Figure 14:**
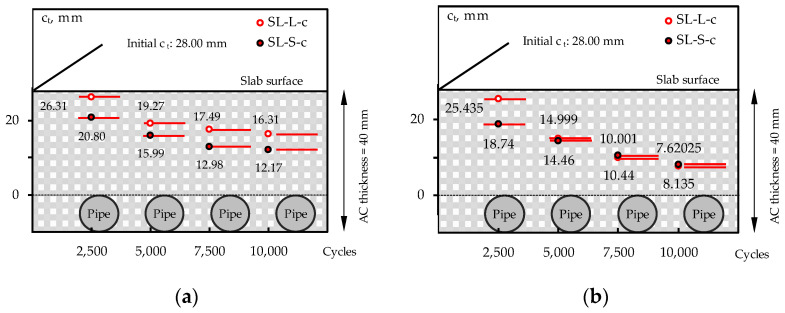
Evolution of covering thickness above the coil: central (**a**) and more-critical (**b**) sections.

Indeed, a further analysis of the experimental results is proposed in Figure 14, where the covering thickness *c_t_* above the coil during the wheel tracking tests is depicted when the metallic body is located in the central slab portion (Figure 14a) or in the most critical section for rutting (Figure 14b). Given the thickness of the upper layer, equal to 40 mm, and the pipe *Φ_N_* of 12 mm, the initial covering thickness is equal to 28 mm. In the case of *ACL*, at the end of the tests (after 10,000 loading cycles), *c_t_* is equal to 12.17 mm and 8.14 mm at the central and the more-deformed sections, respectively. These *c_t_* values are not fully consistent with the typical asphalt layer thickness (20 mm can be considered a sort of minimum threshold limit), the common compaction operations, or the selected NMAS (needed thickness at least three or four times the NMAS) [42,43,44]. Similar results were also found in the case of *ACS* (*SL_S_c*).

Overall, specific improvements to mitigate the rutting potential of the investigated limestone or steel slag-based mixtures are strongly suggested. As a consequence, the thermal properties of the newly designed ASC systems should be reconsidered. Given the need of the reduced 8-mm NMAS to limit the layer thickness in order to guarantee the thermal efficiency of the system, the lithic skeleton can be firstly redesigned; in this sense, ad hoc granulometric distributions should be taken into account to limit the loss of materials, the densification, and the plastic flow mechanisms responsible for rutting [7], thus, also to improve the interlocking effects and promote stone–stone contact [15]. Analogously, mixture optimization against rutting could also be realized by using polymer-modified asphalt binders [41].

## 5. Conclusions and Further Studies

The paper presented an experimental study aimed at evaluating responses in terms of rutting potential of bituminous mixtures manufactured with conventional limestone aggregates or steel slags. Such mixes were designed in previous research with the objective to produce specific asphalt layers for a solar collector system able to harvest thermal energy due to solar irradiation. Based on the promising thermal-related results collected from the previous study, the asphalt concretes were reproduced maintaining the original design: the obtained bituminous mixes (double-layer specimens) were analyzed in the laboratory though a mixture-scale investigation mainly addressed at rutting-related concerns. The main findings can be summarized as follows:

- in terms of visco-elastic properties, steel slag led to an appreciable stiffening effect and a slightly higher viscous behavior with respect to reference bituminous mixtures containing natural limestone aggregate only. The double-layer samples containing the solar collector were always stiffer than the corresponding standard double-layer structure. This result was initially supposed to be beneficial in terms of permanent deformation resistance;
- as expected, permanent deformation test results were in accordance with what was found in terms of stiffness (i.e., the higher the stiffness, the lower the permanent deformation of the materials);
- despite a relatively superficial installation of the collector, which was placed only 28 mm under the surface based on thermal optimization, no differential rutting influenced by the metallic elements was detected during wheel tracking tests. This was verified both for limestone- and slag-based asphalt mixes;
- in absolute terms, the final rut depths produced on the slab surfaces (both in the case of limestone and steel slag) did not match the minimum technical requirements related to rutting resistance; in the same way, the final covering thickness of asphalt above the collector was unacceptable because of the excessive rutting;
- thus, specific suggestions were provided to redesign the mixes towards more rutting-resistant structures while maintain thermal efficiency; in this sense, the optimization of the aggregate gradations and the binder content along with the use of polymer-modified bitumen were identified as possible improvements.

Overall, the present research provides promising elements concerning the possible reuse of steel slag aggregates to enhance anti-rutting properties. Further efforts have been planned to solve the above-mentioned criticisms related to material resistance against rutting; in this sense, the re-design of the asphalt mixtures will also imply a further thermal re-optimization of the asphalt solar collector. Then, real-scale pilot applications could be constructed in order to validate, under actual traffic and environmental conditions, the mechanical and thermal characteristics of the investigated ASC while verifying the available friction properties of the steel-slag based surfaces.

## Figures and Tables

**Table 1:** Physical properties of bitumen utilized for mixtures.

Characteristic	Test Method	Unit	Value
Type	-	-	Non-modified
Penetration at 25 °C	EN 1426	0.1 mm	53
Softening point	EN 1427	°C	50.0
Retained penetration at 25 °C after RTFO	EN 1426	%	70
Increase in softening point after RTFO	EN 1427	°C	6.6
Ductility at 25 °C	ASTM D113	cm	>100

**Table 2:** Mix design of the produced asphalt mixtures.

Constituent	Dosages, % (by Weight) ^1^	
	*ACL*	*ACS*
L 4/8	45.0	-
S 4/8	-	42.0
L 0/4	52.0	-
S 0/4	-	51.0
L filler	3.0	7.0
B content (by aggregate weight), %	5.7	5.9
Target voids, %	4.00	4.00

^1^ Executed volumetric mix-design accounting for different particle density weights of *L* and *S*.

**Table 3:** Details about the produced samples.

Code	Shape	Dimension	Procedure	Lower Layer		Collector	Upper Layer	
				Mix	h, mm		Mix	h, mm
PR_L *	PR ^a^	50 × 400 mm	RC + saw	*ACL*	10	-	*ACL*	40
PR_S_p				*ACL*	10	Pipe ^1^	*ACL*	40
PR_S *				*ACS*	10	-	*ACS*	40
PR_S_p				*ACS*	10	Pipe ^1^	*ACS*	40
CY_L *	CY ^b^	150-mm diam.	SGC	*ACL*	10	-	*ACL*	40
CY_S_p				*ACL*	10	Pipe ^2^	*ACL*	40
CY_S *				*ACS*	10	-	*ACS*	40
CY_S_p				*ACS*	10	Pipe ^2^	*ACS*	40
SL_L *	SL ^c^	300 × 400 mm	RC	*ACL*	10	-	*ACL*	40
SL_S_c				*ACL*	10	Coil	*ACL*	40
SL_S *				*ACS*	10	-	*ACS*	40
SL_S_c				*ACS*	10	Coil	*ACS*	40

* Reference (without ASC); ^a^ prismatic (PR); ^b^ cylindrical (CY); ^c^ slab (SL); ^1^ straight pipe (400-mm long); ^2^ straight pipe (150-mm long).

**Table 4:** Numerical RLA test results (60 °C).

Property	Stress	*CY_L*	*CY_S*	*CY_L_p*	*CY_S-P*
*f_c_*, mm/cycle	300 kPa	4.33	3.655	2.683	1.091
	350 kPa	7.214	6.286	5.176	3.828
	400 kPa	9.864	7.011	6.939	5.456
*k*_1_, -	300 kPa	7033	6193	5788	4506
	350 kPa	7847	9161	6719	5940
	400 kPa	12,367	11,639	20,928	9786
*k*_2_, -	300 kPa	0.139	0.151	0.16	0.162
	350 kPa	0.143	0.136	0.15	0.165
	400 kPa	0.108	0.101	0.109	0.116
*FN*, cycles	300 kPa	2875	3162	3317	4675
	350 kPa	1965	2098	2245	2813
	400 kPa	854	977	1109	2579

**Table 5:** Wheel track results on central slab points: evaluation of creep slopes and final rut depths.

Property		*SL_L*	*SL_S*	*SL_L_c*	*SL_S_c*
1st phase	*f_w_*, mm/cycle	2.20 × 10^−3^	2.48 × 10^−3^	1.05 × 10^−3^	2.91 × 10^−3^
	*R* ^2^	0.93	0.98	0.88	0.95
2nd phase	*f_w_*, mm/cycle	1.02 × 10^−3^	2.02 × 10^−3^	9.93 × 10^−4^	1.14 × 10^−3^
	*R* ^2^	0.98	0.99	0.95	0.99
3rd phase	*f_w_*, mm/cycle	2.53 × 10^−4^	2.48 × 10^−4^	1.29 × 10^−4^	2.61 × 10^−4^
	*R* ^2^	0.86	0.91	0.84	0.98
Final rut depth, mm		17.146	18.447	15.601	15.832

## Data Availability

The data presented in this study are available on request from the corresponding author. The data are not publicly available because they are a part of on-going research.
